# Supplementary material for: Prognostic role of the prognostic nutritional index (PNI) in patients with head and neck neoplasms undergoing radiotherapy: A meta-analysis
Source: PLoS One. 2021 Sep 14;16(9):e0257425. doi: 10.1371/journal.pone.0257425 (PMC8439446; doi:10.1371/journal.pone.0257425)
Supplement: S1 File — (PDF) [file pone.0257425.s001.pdf]

Electronic search strategy：

PubMed

#1 ((((((((((((((((((((((Neoplasms, Head and Neck) OR (Head, Neck Neoplasms)) OR (Cancer of Head and Neck)) OR (Head and Neck Cancer)) OR (Cancer of the Head and Neck)) OR (Upper Aerodigestive Tract Neoplasms)) OR (UADT Neoplasms)) OR (Neoplasm, UADT)) OR (Neoplasms, UADT)) OR (UADT Neoplasm)) OR (Neoplasms, Upper Aerodigestive Tract)) OR (Head Neoplasms)) OR (Neoplasms, Head)) OR (Neck Neoplasms)) OR (Neoplasms, Neck)) OR (Cancer of Head)) OR (Head Cancer)) OR (Cancer of the Head)) OR (Cancer of Neck)) OR (Neck Cancer)) OR (Cancer of the Neck)) OR ("Head and Neck Neoplasms"[Mesh]))

#2 (radiotherapy)

#3(((((((((((((Prognostic Nutritional Index (PNI)) OR (Index, Prognostic Nutritional (PNI))) OR (Indices, Prognostic Nutritional (PNI))) OR (Nutritional Index, Prognostic (PNI))) OR (Nutritional Indices, Prognostic (PNI))) OR (Prognostic Nutritional Indices (PNI))) OR (Prognostic Nutritional Index)) OR (Index, Prognostic Nutritional)) OR (Indices, Prognostic Nutritional)) OR (Nutritional Index, Prognostic)) OR (Nutritional Indices, Prognostic)) OR (Prognostic Nutritional Indices)) OR ("Nutrition Assessment"[Mesh]))

#1 AND #2 AND #3

Web of Science

#1 (Head and Neck Neoplasms) OR (Neoplasms, Head and Neck) OR (Head, Neck Neoplasms) OR (Cancer of Head and Neck) OR (Head and Neck Cancer) OR (Cancer of the Head and Neck) OR (Upper Aerodigestive Tract Neoplasms) OR (UADT Neoplasms) OR (Neoplasm, UADT) OR (Neoplasms, UADT) OR (UADT Neoplasm) OR (Neoplasms, Upper Aerodigestive Tract) OR (Head Neoplasms) OR (Neoplasms, Head) OR (Neck Neoplasms) OR (Neoplasms, Neck) OR (Cancer of Head) OR (Head Cancer) OR (Cancer of the Head) OR (Cancer of Neck) OR (Neck Cancer) OR (Cancer of the Neck)

#2 (Prognostic Nutritional Index (PNI)) OR (Index, Prognostic Nutritional (PNI)) OR (Indices, Prognostic Nutritional (PNI)) OR (Nutritional Index, Prognostic (PNI)) OR (Nutritional Indices, Prognostic (PNI)) OR (Prognostic Nutritional Indices (PNI)) OR (Prognostic Nutritional Index) OR (Index, Prognostic Nutritional) OR (Indices, Prognostic Nutritional) OR (Nutritional Index, Prognostic) OR (Nutritional Indices, Prognostic) OR (Prognostic Nutritional Indices)

#3 radiotherapy

#1 AND #2 AND #3

Embase

#1 (Head and Neck Neoplasms) OR (Neoplasms, Head and Neck) OR (Head, Neck Neoplasms) OR (Cancer of Head and Neck) OR (Head and Neck Cancer) OR (Cancer of the Head and Neck) OR (Upper Aerodigestive Tract Neoplasms) OR (UADT Neoplasms) OR (Neoplasm, UADT) OR (Neoplasms, UADT) OR (UADT Neoplasm) OR (Neoplasms, Upper Aerodigestive Tract) OR (Head Neoplasms) OR (Neoplasms, Head) OR (Neck Neoplasms) OR (Neoplasms, Neck) OR (Cancer of Head) OR (Head Cancer) OR (Cancer of the Head) OR (Cancer of Neck) OR (Neck Cancer) OR (Cancer of the Neck)

#2 (Prognostic Nutritional Index (PNI)) OR (Index, Prognostic Nutritional (PNI)) OR (Indices, Prognostic Nutritional (PNI)) OR (Nutritional Index, Prognostic (PNI)) OR (Nutritional Indices, Prognostic (PNI)) OR (Prognostic Nutritional Indices (PNI)) OR (Prognostic Nutritional Index) OR (Index, Prognostic Nutritional) OR (Indices, Prognostic Nutritional) OR (Nutritional Index, Prognostic) OR (Nutritional Indices, Prognostic) OR (Prognostic Nutritional Indices)
#3 'radiotherapy'/exp
#1 AND #2 AND #3

When we conducted literature retrieval by MeSH plus with entry terms systematically, we chose expanded search at the same time, which means it included MeSH terms found below this targeted term in the MeSH hierarchy. That is how we ensure that we can retain all of the relevant articles including all kinds of sub types of head and neck neoplasms.

On top of that, we also traced citations in the reference sections of the retrieved studies so that to make sure no one of eligible studies were left.
